# Supplementary material for: Genome-wide identification of the MIOX gene family and their expression profile in cotton development and response to abiotic stress
Source: PLoS One. 2021 Jul 9;16(7):e0254111. doi: 10.1371/journal.pone.0254111 (PMC8270170; doi:10.1371/journal.pone.0254111)
Supplement: S1 Fig — Multiple sequence alignments were conducted using ClustalW. (PDF) [file pone.0254111.s001.pdf]

```
GdMIOX01     ............................................................
GtMIOX01     ............................................................
GbMIOX01     ............................................................
GaMIOX01     ............................................................
GkkMIOX01    ............................................................
GhMIOX01     ............................................................
GmMIOX01     ............................................................
GherMIOX01   ............................................................
GbMIOX07     ............................................................
GtMIOX06     ............................................................
GrMIOX01     ............................................................
GhMIOX07     ............................................................
GdMIOX06     ............................................................
GmMIOX06     ............................................................
GmMIOX05     ............................................................
GtMIOX05     ............................................................
GdMIOX05     ............................................................
GaMIOX05     ............................................................
GbMIOX06     ............................................................
GhMIOX06     ............................................................
GhMIOX12     ............................................................
GtMIOX11     ............................................................
GbMIOX12     ............................................................
GrMIOX03     ............................................................
GherMIOX06   ............................................................
GdMIOX11     ............................................................
GmMIOX11     ............................................................
GkkMIOX05    ............................................................
ATMIOX03     ............................................................
ATMIOX04     ............................................................
GaMIOX03     ............................................................
GdMIOX03     ............................................................
GherMIOX03   ............................................................
GbMIOX03     ............................................................
GtMIOX03     ............................................................
GhMIOX03     ............................................................
GmMIOX03     ............................................................
GkkMIOX03    ............................................................
GtMIOX08     ............................................................
GbMIOX09     ............................................................
GdMIOX08     ............................................................
GhMIOX09     ............................................................
GrMIOX05     ............................................................
GmMIOX08     ............................................................
ATMIOX02     ............................................................
GherMIOX04   ............................................................
GhMIOX04     ............................................................
GaMIOX04     ............................................................
GbMIOX04     ............................................................
GtMIOX04     ............................................................
GmMIOX04     ............................................................
GdMIOX09     ............................................................
GtMIOX09     ............................................................
GbMIOX10     ............................................................
GhMIOX10     ............................................................
GmMIOX09     ............................................................
GrMIOX06     ............................................................
GdMIOX04     ............................................................
GkkMIOX04    ............................................................
GhMIOX02     ............................................................
GtMIOX02     ............................................................
GbMIOX02     ............................................................
GmMIOX02     ............................................................
GaMIOX02     ............................................................
GhMIOX08     ............................................................
GrMIOX04     ............................................................
GbMIOX08     ............................................................
GdMIOX07     ............................................................
GtMIOX07     ............................................................
GmMIOX07     ............................................................
GherMIOX02   ............................................................
GkkMIOX02    ............................................................
GdMIOX02     ............................................................
ATMIOX01     ............................................................
GaMIOX06     ............................................................
GhMIOX05     ............................................................
GbMIOX11     ............................................................
GdMIOX10     ............................................................
GtMIOX10     ............................................................
GrMIOX02     ............................................................
GhMIOX11     ............................................................
GmMIOX10     ............................................................
GbMIOX05     ............................................................
GherMIOX05   MEGYRVDEAVIALKLKDASNWWHDINESTLWQDRIFHILAALYGLVAAVALVQLIRIQLR
GkkMIOX06    ............................................................
```

```
GdMIOX01     ............................................................
GtMIOX01     ............................................................
GbMIOX01     ............................................................
GaMIOX01     ............................................................
GkkMIOX01    ............................................................
GhMIOX01     ............................................................
GmMIOX01     ............................................................
GherMIOX01   ............................................................
GbMIOX07     ............................................................
GtMIOX06     ............................................................
GrMIOX01     ............................................................
GhMIOX07     ............................................................
GdMIOX06     ............................................................
GmMIOX06     ............................................................
GmMIOX05     ............................................................
GtMIOX05     ............................................................
GdMIOX05     ............................................................
GaMIOX05     ............................................................
GbMIOX06     ............................................................
GhMIOX06     ............................................................
GhMIOX12     ............................................................
GtMIOX11     ............................................................
GbMIOX12     ............................................................
GrMIOX03     ............................................................
GherMIOX06   ............................................................
GdMIOX11     ............................................................
GmMIOX11     ............................................................
GkkMIOX05    ............................................................
ATMIOX03     ............................................................
ATMIOX04     ............................................................
GaMIOX03     ............................................................
GdMIOX03     ............................................................
GherMIOX03   ............................................................
GbMIOX03     ............................................................
GtMIOX03     ............................................................
GhMIOX03     ............................................................
GmMIOX03     ............................................................
GkkMIOX03    ............................................................
GtMIOX08     ............................................................
GbMIOX09     ............................................................
GdMIOX08     ............................................................
GhMIOX09     ............................................................
GrMIOX05     ............................................................
GmMIOX08     ............................................................
ATMIOX02     ............................................................
GherMIOX04   ............................................................
GhMIOX04     ............................................................
GaMIOX04     ............................................................
GbMIOX04     ............................................................
GtMIOX04     ............................................................
GmMIOX04     ............................................................
GdMIOX09     ............................................................
GtMIOX09     ............................................................
GbMIOX10     ............................................................
GhMIOX10     ............................................................
GmMIOX09     ............................................................
GrMIOX06     ............................................................
GdMIOX04     ............................................................
GkkMIOX04    ............................................................
GhMIOX02     ............................................................
GtMIOX02     ............................................................
GbMIOX02     ............................................................
GmMIOX02     ............................................................
GaMIOX02     ............................................................
GhMIOX08     ............................................................
GrMIOX04     ............................................................
GbMIOX08     ............................................................
GdMIOX07     ............................................................
GtMIOX07     ............................................................
GmMIOX07     ............................................................
GherMIOX02   ............................................................
GkkMIOX02    ............................................................
GdMIOX02     ............................................................
ATMIOX01     ............................................................
GaMIOX06     ............................................................
GhMIOX05     ............................................................
GbMIOX11     ............................................................
GdMIOX10     ............................................................
GtMIOX10     ............................................................
GrMIOX02     ............................................................
GhMIOX11     ............................................................
GmMIOX10     ............................................................
GbMIOX05     ............................................................
GherMIOX05   VPEYGWTTQKVFHFLNFLVNGVRGLVFVFRRDVQNLHPEIVQHILLDMPSLAFFTTYALL
GkkMIOX06    ............................................................
```

```
GdMIOX01     ............................................................
GtMIOX01     ............................................................
GbMIOX01     ............................................................
GaMIOX01     ............................................................
GkkMIOX01    ............................................................
GhMIOX01     ............................................................
GmMIOX01     ............................................................
GherMIOX01   ............................................................
GbMIOX07     ............................................................
GtMIOX06     ............................................................
GrMIOX01     ............................................................
GhMIOX07     ............................................................
GdMIOX06     ............................................................
GmMIOX06     ............................................................
GmMIOX05     ............................................................
GtMIOX05     ............................................................
GdMIOX05     ............................................................
GaMIOX05     ............................................................
GbMIOX06     ............................................................
GhMIOX06     ............................................................
GhMIOX12     ............................................................
GtMIOX11     ............................................................
GbMIOX12     ............................................................
GrMIOX03     ............................................................
GherMIOX06   ............................................................
GdMIOX11     ............................................................
GmMIOX11     ............................................................
GkkMIOX05    ............................................................
ATMIOX03     ............................................................
ATMIOX04     ............................................................
GaMIOX03     ............................................................
GdMIOX03     ............................................................
GherMIOX03   ............................................................
GbMIOX03     ............................................................
GtMIOX03     ............................................................
GhMIOX03     ............................................................
GmMIOX03     ............................................................
GkkMIOX03    ............................................................
GtMIOX08     ............................................................
GbMIOX09     ............................................................
GdMIOX08     ............................................................
GhMIOX09     ............................................................
GrMIOX05     ............................................................
GmMIOX08     ............................................................
ATMIOX02     ............................................................
GherMIOX04   ............................................................
GhMIOX04     ............................................................
GaMIOX04     ............................................................
GbMIOX04     ............................................................
GtMIOX04     ............................................................
GmMIOX04     ............................................................
GdMIOX09     ............................................................
GtMIOX09     ............................................................
GbMIOX10     ............................................................
GhMIOX10     ............................................................
GmMIOX09     ............................................................
GrMIOX06     ............................................................
GdMIOX04     ............................................................
GkkMIOX04    ............................................................
GhMIOX02     ............................................................
GtMIOX02     ............................................................
GbMIOX02     ............................................................
GmMIOX02     ............................................................
GaMIOX02     ............................................................
GhMIOX08     ............................................................
GrMIOX04     ............................................................
GbMIOX08     ............................................................
GdMIOX07     ............................................................
GtMIOX07     ............................................................
GmMIOX07     ............................................................
GherMIOX02   ............................................................
GkkMIOX02    ............................................................
GdMIOX02     ............................................................
ATMIOX01     ............................................................
GaMIOX06     ....................................MTILIDQP................
GhMIOX05     ....................................MTILIDQPDFGEFLFVSSINDLVL
GbMIOX11     ............................................................
GdMIOX10     ....................................MTILIDQP................
GtMIOX10     ....................................MTILIDQP................
GrMIOX02     ....................................MTILIDQP................
GhMIOX11     ....................................MTILIDQP................
GmMIOX10     ....................................MILFFFFD................
GbMIOX05     ............................................................
GherMIOX05   VLFWAEIYYQARAVSTDGLRPSFFTINAVVYTIQIGMWLILWWKYIPVLVVLSKVFFAGV
GkkMIOX06    ............................................................
```

```
                            1        10        20
GdMIOX01      ................MVTICIGKVDQAKGQKIQLD........................
GtMIOX01      ................MVTICIGKVDQAKGQKIQLD........................
GbMIOX01      ..MSFSFFFRFFSRILQHFYDEGPIDQAKGQKIQLD........................
GaMIOX01      ..MSFSFFFRFFSRILQHFYDGGPIDQAKGQKIQLD........................
GkkMIOX01     .................MSFTWCLVDQAKGQKIQVD........................
GhMIOX01      ................MVTICIGKVDQAKGQKIQLD........................
GmMIOX01      ................MVTICIGKVDQAKGQKIQLD........................
GherMIOX01    ................MVTICIGKVDQAKGQKIQLD........................
GbMIOX07      ................MVTICIGKVGQAKGQKIQLD........................
GtMIOX06      ................MVTICIGKVGQAKGQKIQLD........................
GrMIOX01      ................MVTICIGKVGQAKGQKIQLD........................
GhMIOX07      ..................MTRIIHIGQAKGQKIQLD........................
GdMIOX06      ............................................................
GmMIOX06      ............................................................
GmMIOX05      ..............MTVSVEKPNVTQAKSAEQAQPD........................
GtMIOX05      ..............MTVSVEKPNVTQAKSAEQALPD........................
GdMIOX05      ..............MTVSVEKPNVTQAKSAEQAQPD........................
GaMIOX05      ..............MTVSVEKPNVTQAKSAEQAQPD........................
GbMIOX06      ..............MTVSVEKP...NAKSAEQAQPD........................
GhMIOX06      ..............MTVSVEKP...NAKSAEQAQPD........................
GhMIOX12      ..............MIVSVEKPNATQAKSAEQAQPD........................
GtMIOX11      ..............MIVSVEKPNATQAKSAEQAQPD........................
GbMIOX12      ..............MIVSVEKPNATQAKSAEQAQPD........................
GrMIOX03      ..............MIVSVEKPNATQAKSAEQAQPD........................
GherMIOX06    ..............MTVSVEKPNVTQAKSAEQAQPD........................
GdMIOX11      ............................................................
GmMIOX11      ............................................................
GkkMIOX05     ............................................................
ATMIOX03      .MTISVEKPIFEEVSAFEKSGDNIGELKLDGGFSMP........................
ATMIOX04      .MNISVENPVFVHEDSTTQKTG...ELRLDSDIPMS........................
GaMIOX03      ..MTILIEKP....ELDCQIHVDE.SKELVLDGGFP........................
GdMIOX03      ..MTILIEKP....ELDCQIHVDE.SKELVLDGGFP........................
GherMIOX03    ..MTILIEKP....ELDCQIHVDE.SKELVLDGGFP........................
GbMIOX03      ..MTILIEKPELVSQSDCQIHVDE.SKELVLDGGFP........................
GtMIOX03      ..MTILIEKPELVSQSDCQIHVDE.SKELVLDGGFP........................
GhMIOX03      ..MTILIEKPELVSQSDCQIHVDE.SKELVLDGGFP........................
GmMIOX03      ..MTILIEKPELVSQSDCQIHVDE.SKELVLDGGFP........................
GkkMIOX03     ..MTILIEKPELVFQPDCQNHVDE.SKELVLDGGFP........................
GtMIOX08      ..MTILMEKPELVSQSDCQNHVDE.SKELVLDDGFP........................
GbMIOX09      ..MTILMEKPELVSQSDCQNHVDE.SKELVLDGGFP........................
GdMIOX08      ..MTILMEKPELVSQSDCQNHVDE.SKELVLDGGFP........................
GhMIOX09      ..MTILMEKPELVSQSDCQNHVDE.SKELVLDGGFP........................
GrMIOX05      ..MTILMEKPELVSQSDFQNHADE.SKELVLDGGFP........................
GmMIOX08      ............................................................
ATMIOX02      ..MTILVEHFVPDSRVDEKKVIEERDNELVLDGGFV........................
GherMIOX04    ..............MTILIDQPDFGIEAGFNKADDV........................
GhMIOX04      ..............MTILIDQPDFGIEAGFNKADDV........................
GaMIOX04      ..............MTILIDQPDFGIEAGFNKADDV........................
GbMIOX04      ..............MTILIDQPDFGIEAGFNKADDV........................
GtMIOX04      ..............MTILIDQPDFGIEAGFNKADDV........................
GmMIOX04      ..............MTILIDQPDFGIEAGFNKADDV........................
GdMIOX09      ..............MTILIDQPDFGIEAGFNKADDV........................
GtMIOX09      ..............MTILIDQPDFGIEAGFNKADDV........................
GbMIOX10      ..............MTILIDQPDFGIEAGFNKADDV........................
GhMIOX10      ..............MTILIDQLDFGIEAGFNKADDV........................
GmMIOX09      ..............MTILIDQPDFGIEAGFNKADDV........................
GrMIOX06      ..............MTILIDQPDFGIEAGFNKADDV........................
GdMIOX04      ............................................................
GkkMIOX04     ............................................................
GhMIOX02      ..............MTILIDQPELGVEAGVNKLDNA........................
GtMIOX02      ..............MTILIDQPELGVEAGVNKLDNA........................
GbMIOX02      ..............MTILIDQPELGVEAGVNKLDNA........................
GmMIOX02      ..............MTILIDQPELGVEAGVNKLDNA........................
GaMIOX02      ..............MTILIDQPELGVEAGVNKLDNA........................
GhMIOX08      ..............MTILIDQPELGVEAGVNKLDNA........................
GrMIOX04      ..............MTILIDQPELGVEAGVNKLDNA........................
GbMIOX08      ..............MTILIDQPELGVEAGVNKLDNA........................
GdMIOX07      ..............MTILIDQPELGVEAGVNKLDNA........................
GtMIOX07      ..............MTILIDQPELGVEAGVNKLDNA........................
GmMIOX07      ..............MTILIDQPELGVEAGVNKLDNA........................
GherMIOX02    ..............MTILIDQPELGVEAGVNKLDNA........................
GkkMIOX02     ............................................................
GdMIOX02      ............................................................
ATMIOX01      .......MTILIDRHSDQNDAGDEIVEKNQGNGKEE........................
GaMIOX06      ....................DFEAGIEERHKVAVDE........................
GhMIOX05      GVHFFDTFCCLYYDSFFFFDDIEAGIEERHKVAVDE........................
GbMIOX11      ............................................................
GdMIOX10      ....................DFEAGIEERHKVAIDE........................
GtMIOX10      ....................DFEAGIEERHKVAIDE........................
GrMIOX02      ....................DFEAGIEERHKVAIDE........................
GhMIOX11      ....................DFEARIEERHKVAIDE........................
GmMIOX10      ....................DIEAGIEERHKVATDE........................
GbMIOX05      ............................................................
GherMIOX05    SLFAALGFLLYGGRLFLMLQRFPVESKGRRKKLQEVGYVTTICFLCFLVRCIMMCFNAFD
GkkMIOX06     ............................................................
```

```
                                                30        40                 50
GdMIOX01     ..................ANELVSNDEFALPENNAFGN....SFRDYEADNGRKDIVEQH
GtMIOX01     ..................ANELVSNDEFALPENNAFGN....SFRDYEADNGRKDIVEQH
GbMIOX01     ..................ANELVSNDEFALPENNAFGN....SFRDYEADNGRKDIVEQH
GaMIOX01     ..................ANELVSNDEFALPEKNAFGN....SFRDYEADNGRKDIVEQH
GkkMIOX01    ..................ANELVSNDEFALPEKNAFGN....SFRDYEADNGRKDIVEQH
GhMIOX01     ..................ANELVSNDEFALPENNAFGN....SFRDYEADNGRKDIVEQH
GmMIOX01     ..................VNELVSNDEFALPENNAFGN....SFRDYEADNGRKDIVEQH
GherMIOX01   ..................ANELVSNDEFALPEKNAFGN....SFRDYEADNGRKDIVEQH
GbMIOX07     ..................ANELVSNDEFALPEKNAFGN....SFRDYETDNGRKDIVEQH
GtMIOX06     ..................ANELVSNDEFALPEKNAFGN....SFRDYETDNGRKDIVEQH
GrMIOX01     ..................ANELVSNDEFALPEKNAFGN....SFRDYETDNGRKDIVEQH
GhMIOX07     ..................ANELVSNDEFALPEKNAFGNSLIYIHMDYETDNGRKDIVEQH
GdMIOX06     ............................................................
GmMIOX06     ............................................................
GmMIOX05     ..................ANKLVSNDQFTVPQNNAFGN.....TFRDYEVGARTDKVEQH
GtMIOX05     ..................ANKLVSNDQFTVPQNNAFGN.....TFRDYEVGAR.......
GdMIOX05     ..................ANKLVSNDQFTVPQNNAFGN.....TFRDYEVGARTDKVEQH
GaMIOX05     ..................ANKLVSNDQFTVPQNNAFGN.....TFRDYEVGARTDKVEQH
GbMIOX06     ..................ANKLVSNDQFTVPQNNAFGN.....TFRDYEVGARTDKVEQH
GhMIOX06     ..................ANKLVSNDQFTVPQNNAFGN.....TFRDYEVGARTDKVEQH
GhMIOX12     ..................ANKLVSNDQFTVPQNNAFGN.....TFRDYEVGARTDKVEQH
GtMIOX11     ..................ANKLVSNDQFTVPQNNAFGN.....TFRDYEVGARTDKVEQH
GbMIOX12     ..................ANKLVSNDQFTVPQNNAFGN.....TFRDYEVGARTDKVEQH
GrMIOX03     ..................ANKLVSNDQFTVPQNNAFGN.....KFRDYEVGARTDKVEQH
GherMIOX06   ..................ANKLVSNDQFTVPQNNAFGN.....TFRDYEVGARTDKVEQH
GdMIOX11     ............................................................
GmMIOX11     ............................................................
GkkMIOX05    ............................................................
ATMIOX03     ..................KMDTNDDEAFLAPEMNAFGR...QFRDYDVE.SERQKGVEEF
ATMIOX04     ..................KISS.DDEVFLAPEMNAFGR...QFRDYTDTNSERQKSVEHF
GaMIOX03     ..................VPKSLSGEGFLAPEVNSFGN...SFRDYN.AESERQKSVEEF
GdMIOX03     ..................VPKSLSGEGFLAPEVNSFGN...SFRDYN.AESERQKSVEEF
GherMIOX03   ..................VPKSLSGEGFLAPEVNSFGN...SFRDYN.AESERQKSVEEF
GbMIOX03     ..................VPKSLSGEGFLAPEVNSFGN...SFRDYN.AESERQKSVEEF
GtMIOX03     ..................VPKSLSGEGFLAPEVNSFGN...SFRDYN.AESERQKSVEEF
GhMIOX03     ..................VPKSLSGEGFLAPEVNSFGN...SFRDYN.AESERQKSVEEF
GmMIOX03     ..................VPKSLSGEGFLAPEVNSFGN...SFRDYN.AESERQKSVEEF
GkkMIOX03    ..................VPKSLSGEGFLAPEINSFGN...SFRDYN.AESERQKSVEEF
GtMIOX08     ..................VPKSLSGEGFLAPEINSFGN...SFRDYN.AESERQKSVEEF
GbMIOX09     ..................VPKSLSGEGFLAPEINSFGN...SFRDYN.AESERQKSVEEF
GdMIOX08     ..................VPKSLSGEGFLAPEINSFGN...SFRDYN.AESERQKSVEEF
GhMIOX09     ..................VPKSLSGEGFLAPEINSFGN...SFRDYN.AESERQKSVEEF
GrMIOX05     ..................VPKSLSGEGFLAPEINSFGN...SFRDYN.AESERQKSVEEF
GmMIOX08     ............................................................
ATMIOX02     ..................VPKSKETDAFDAPDMNFLGH...SFRDYENGESERQQGVEEF
GherMIOX04   ..................EKEGVLNGGFMMPHTNSFGHT....FRDYHVESERQQGVENF
GhMIOX04     ..................EKEGVLNGGFMMPHTNSFGHT....FRDYHVESERQQGVENF
GaMIOX04     ..................EKEGVLNGGFMMPHTNSFGHT....FRDYHVESERQQGVENF
GbMIOX04     ..................EKEGVLNGGFMMPHTNSFGHT....FRDYHVESERQQGVENF
GtMIOX04     ..................EKEGVLNGGFMMPHTNSFGHT....FRDYHVESERQQGVENF
GmMIOX04     ..................EKEGVLNGGFMMPHTNSFGHT....FRDYHVESERQQGVENF
GdMIOX09     ..................EKEAVLNGGFMMPHTNSFGHT....FRDYHVESERQQGVENF
GtMIOX09     ..................EKEAVLNGGFMMPHTNSFGHT....FRDYHVESERQQGVENF
GbMIOX10     ..................EKEAVLNGGFMMPHTNSFGHT....FRDYHVESERQQGVENF
GhMIOX10     ..................EKEAVLNGGFMMPHTNSFGHT....FRDYHVESERQQGVENF
GmMIOX09     ..................EKEGVLHGGFMMPHTNSFGHT....FRDYHVESERQQGVENF
GrMIOX06     ..................EKEGVLHGGFMMPHTNSFGHT....FRDYHVESERQQGVETF
GdMIOX04     ............................MMPHTNSFGHT....FRDYHVESERQQGVENF
GkkMIOX04    ............................................................
GhMIOX02     ..................DNELVLDGGFVVPQTNSFGHT....FRDYHVESERQQGVENF
GtMIOX02     ..................DNELVLDGGFVVPQTNSFGHT....FRDYHVESERQQGVENF
GbMIOX02     ..................DNELVLDGGFVVPQTNSFGHT....FRDYHVESERQQGVENF
GmMIOX02     ..................DNELVLDGGFVVPQTNSFGHT....FRDYHVESERQQGVENF
GaMIOX02     ..................DNELVLDGGFVVPQTNSFGHT....FRDYHVESERQQGVENF
GhMIOX08     ..................DNELVLDGGFVVPQTNSFGQT....FRDYHVESERQQGVENF
GrMIOX04     ..................DNELVLDGGFVVPQTNSFGHT....FRDYHVESERQQGVENF
GbMIOX08     ..................DNELVLDGGFVVPQTNSFGHT....FRDYHVESERQQGVENF
GdMIOX07     ..................DNELVLDGGFVVPQTNSFGHT....FRDYHVESERQQGVENF
GtMIOX07     ..................DNELVLDGGFVVPQTNSFGHT....FRDYHVESERQQGVENF
GmMIOX07     ..................DNELVLDGGFVVPQTNSFGHT....FRDYHVESERQQGVENF
GherMIOX02   ..................DNELVLDGGFVVPQTNSFGHT....FRDYHVESERQQGVENF
GkkMIOX02    ............................................................
GdMIOX02     ............................................................
ATMIOX01     ..................ETELVLDAGFEAPHTNSFGRT....FRDYDAESERRRGVEEF
GaMIOX06     ..................NELKTSDGGFVAPHINSFGH....NFR..........ELEEF
GhMIOX05     ..................NELKTSDGGFVAPHINSFGH....NFR..........ELEEF
GbMIOX11     ............................................................
GdMIOX10     ..................NELKTSDGGFVAPHINSFGH....NFRDYHVQSERQKGVEEF
GtMIOX10     ..................NELKTSDGGFVVPHINSFGH....NFRDYHVQSERQKGVEEF
GrMIOX02     ..................NELKTFDGGFVAPHINSFGH....NFRDYHVESERQKGVEEF
GhMIOX11     ..................NELKTSDGGFVAPHINSFGH....NFRDYHVQSERQKGVEEF
GmMIOX10     ..................NELKTSDGGFVAPHINSFGH....NFRDYHVQSERQKGVEEF
GbMIOX05     ............................................................
GherMIOX05   KAADLDVLNHPVLNLIYYLLVEILPSSLVLFILRKLPP....KREIIMWKAKGKRELEEF
GkkMIOX06    ............................................................
```

```
             60        70        80        90       100       110
GdMIOX01     YKSSHINQTYDFVKQMREEYMKLDKAEMGIWECCELLNEVVDDSDPDLDEPQIQHLLQSA
GtMIOX01     YKSSHINQTYDFVKQMREEYMKLDKAEMGIWECCELLNEVVDDSDPDLDEPQIQHLLQSA
GbMIOX01     YKSSHINQTYDFVKQMREEYMKLDKAEMGIWECCELLNEVVDDSDPDLDEPQIQHLLQSA
GaMIOX01     YKSSHINQTYDFVKQMREEYMKLDKAEMGIWECCELLNEVVDDSDPDLDEPQIQHLLQSA
GkkMIOX01    YKSSHIKQTYDFVKQMREEYMKLDKAKMGIWECCELLNEVVDDSDPDLDEPQIQHLLQSA
GhMIOX01     YKSSHINQTYDFVKQMREEYMKLDKAEMGIWECCELLNEVVDDSDPDLDEPQIQHLLQSA
GmMIOX01     YKSSHINQTYDFVKQMREEYMKLDKAEMGIWECCELLNEVVDDSDPDLDEPQIQHLLQSA
GherMIOX01   YKSSHINQTYDFVKQMREEYMKLDKAEMGIWECCELLNEVVDDSDPDLDEPQIQHLLQSA
GbMIOX07     YKSSHINQTYEFVKQMREEYMKLDKAEMGIWECCELLNEIVDESDPDLDEPQIQHLLQSA
GtMIOX06     YKSSHINQTYEFVKQMREEYMKLDKAEMGIWECCELLNEIVDESDPDLDEPQIQHLLQSA
GrMIOX01     YKSSHINQTYEFVKQMREEYMKLDKAEMGIWECCELLNEIVDESDPDLDEPQIQHLLQSA
GhMIOX07     YKSSHINQTYEFVKQMREEYMKLDKAEMGIWECCELLNEIVDESDPDLDEPQIQHLLQSA
GdMIOX06     ....................MKLDKAEMGIWECCELLNEIVDESDPDLDEPQIQHLLQSA
GmMIOX06     ...............MREEYMKLDKAEMGIWECCELLNEIVDESDPDLDEPQIQHLLQSA
GmMIOX05     YKASHINQTYDFVKKIREEYKKLNKAEMGIWECCELLNEVVDDSDPDLDEPQIQHLLQSA
GtMIOX05     .........TDKVKKIREEYKKLNKAEMGIWECCELLNEVVDDSDPDLDEPQIQHLLQSA
GdMIOX05     YKASHINQTYDFVKKIREEYKKLNKAEMGIWECCELLNEVVDDSDPDLDEPQIQHLLQSA
GaMIOX05     YKASHINQTYDFVKKIREEYKKLNKAEMGIWECCELLNEVVDDSDPDLDEPQIQHLLQSA
GbMIOX06     YKASHINQTYDFGRKSLEEYKKLNKAEMGIWECCELLNEVVDDSDPDLDEPQIQKDYPNE
GhMIOX06     YKASHINQTYDFVKKIREEYKKLNKAEMGIWECCELLNEVVDDSDPDLDEPQIQKDYPNE
GhMIOX12     YKASHINQTYDFVKKIREEYKKLSKAEMGIWECCELLNEVVDDSDPDLDEPQIQHLLQSA
GtMIOX11     YKASHINQTYDFVKKIREEYKKLSKAEMGIWECCELLNEVVDDSDPDLDEPQIQHLLQSA
GbMIOX12     YKASHINQTYDFVKKIREEYKKLSKAEMGIWECCELLNEVVDDSDPDLDEPQIQHLLQSA
GrMIOX03     YKASHINQTYDFVKKIREEYKKLNKAEMGIWECCELLNEVVDDSDPDLDEPQIQHLLQSA
GherMIOX06   YK..........VKKIREEYKKLNKAEMGIWECCELLNEVVDDSDPDLDEPQIQHLLQSA
GdMIOX11     ...........................MGIWECCELLNEVVDDSDPDLDEPQIQHLLQSA
GmMIOX11     ...........................MGIWECCELLNEVVDDSDPDLDEPQIQHLLQSA
GkkMIOX05    ...........................MGIWECCELLNEVVDDSDPDLDEPQIQHLLQSA
ATMIOX03     YRLQHINQTVDFVKKMRAEYGKLDKMVMSIWECCELLNEVVDESDPDLDEPQIQHLLQSA
ATMIOX04     YATQHTNQTLDFVQKMRSEYGKLDKMVMNIWECCELSKEVVDESDPDLDEPQIQHLLQSA
GaMIOX03     YKQQHVNQTYDFVQKMREEYSKLNRMEMSIWECCELLNEVVDDSDPDLDEPQIQHLLQSA
GdMIOX03     YKQQHVNQTYDFVQKMKEEYSKLNRMEMSIWECCELLNEVVDDSDPDLDEPQIQHLLQSA
GherMIOX03   YKQQHVNQTYDFVQKMREEYSKLNRMEMSIWECCELLNEVVDDSDPDLDEPQIQHLLQSA
GbMIOX03     YKQQHVNQTYDFVQKMKEEYSKLNRMEMSIWECCELLNEVVDDSDPDLDEPQIQHLLQSA
GtMIOX03     YKQQHVNQTYDFVQKMKEEYSKLNRMEMSIWECCELLNEVVDDSDPDLDEPQIQHLLQSA
GhMIOX03     YKQQHVNQTYDFVQKMREEYSKLNRMEMSIWECCELLNEVVDDSDPDLDEPQIQHLLQSA
GmMIOX03     YKQQHVNQTYDFVQKMREEYSKLNRMEMSIWECCELLNEVVDDSDPDLDEPQIQHLLQSA
GkkMIOX03    YKQQHINQTYDFVQKMREEYSKLNRMEMSIWECCELLNEVVDDSDPDLDEPQIQHLLQSA
GtMIOX08     YKQQHVNQTYDFVQKMREEYSKLNRMEMSIWECCELLNEVVDDSDPDLDEPQIQHLLQSA
GbMIOX09     YKQQHVNQTYDFVQKMREEYSKLNRMEMSIWECCELLNEVVDDSDPDLDEPQIQHLLQSA
GdMIOX08     YKQQHVNQTYDFVQKMREEYSKLNRMEMSIWECCELLNEVVDDSDPDLDEPQIQHLLQSA
GhMIOX09     YKQQHVNQTYDFVQKMREEYSKLNRMEMSIWECCELLNEVVDDSDPDLDEPQIQHLLQSA
GrMIOX05     YKQQHVNQTYDFVQKMREEYSKLNRMEMSIWECCELLNEVVDDSDPDLDEPQIQHLLQSA
GmMIOX08     ............................................MRWWMTVTLTWMNLKF
ATMIOX02     YRMQHIHQTYDFVKKMRKEYGKLNKMEMSIWECCELLNNVVDESDPDLDEPQIQHLLQTA
GherMIOX04   YRTNHINQTYDFVKRMREEYGNLDKVEMSIWECCELLNDVVDESDPDLDEPQIEHLLQTA
GhMIOX04     YRTNHINQTYDFVKRMREEYGNLDKVEMSIWECCELLNDVVDESDPDLDEPQIEHLLQTA
GaMIOX04     YRTNHINQTYDFVKRMREEYGNLDKVEMSIWECCELLNDVVDESDPDLDEPQIEHLLQTA
GbMIOX04     YRTNHINQTYDFVKRMREEYGNLDKVEMSIWECCELLNDVVDESDPDLDEPQIEHLLQTA
GtMIOX04     YRTNHINQTYDFVKRMREEYGNLDKVEMSIWECCELLNDVVDESDPDLDEPQIEHLLQTA
GmMIOX04     YRTNHIKQTYDFVKRMREEYGNLDKVEMSIWECCELLNDVVDESDPDLDEPQIEHLLQTA
GdMIOX09     YRTNHINQTYDFVKRMREEYGNLDKVEMSIWECCELLNDVVDESDPDLDEPQIEHLLQTA
GtMIOX09     YRTNHINQTYDFVKRMREEYGNLDKVEMSIWECCELLNDVVDESDPDLDEPQIEHLLQTA
GbMIOX10     YRTNHINQTYDFVKRMREEYGNLDKVEMSIWECCELLNDVVDESDPDLDEPQIEHLLQTA
GhMIOX10     YRTNHINQTYDFVKRMREEYGNLDKVEMSIWECCELLNDVVDESDPDLDEPQIEHLLQTA
GmMIOX09     YRTNHINQTYDFVKRMREEYGNLDKVEMSIWECCELLNDVVDESDPDLDEPQIEHLLQTA
GrMIOX06     YRTNHINQTYDFVKRMREEYGNLDRVEMSIWECCELLNDVVDESDPDLDEPQTEHLLQTA
GdMIOX04     YRTNHINQTYDFVKRMREEYGNLDKVEMSIWECCELLNDVVDESDPDLDEPQIEHLLQTA
GkkMIOX04    ...............MREEYGNLDKVEMSIWECCELLNDVVDESDPDLDEPQIEHLLQTA
GhMIOX02     YRTNHINQTYDFS......IGKLNKMEMGIWECCELLNDVIDESDPDLDEPQIEHLLQTA
GtMIOX02     YRTNHINQTYDFVKRMRTEYGKLNKMEMGIWECCELLNDVIDESDPDLDEPQIEHLLQTA
GbMIOX02     YRTNHINQTYDFVKRMRTEYGKLNKMEMGIWECCELLNDVIDESDPDLDEPQIEHLLQTA
GmMIOX02     YRTNHINQTYDFVKRMRTEYGKLNKMEMGIWECCELLNDVIDESDPDLDEPQIEHLLQTA
GaMIOX02     YRTNHIYQTYDFVKRMRTEYGKLNKMEMGIWECCELLNDVIDESDPDLDEPQIEHLLQTA
GhMIOX08     YRTNHINQTSDFVKRMRTEYGKLNKMEMGIWECCELLNDVIDESDPDLDEPQIEHLLQTA
GrMIOX04     YRTNHINQTSDFVKRMRTEYGKLNKMEMGIWECCELLNDVIDESDPDLDEPQIEHLLQTA
GbMIOX08     YRTNHINQTSDFVKRMRTEYGKLNKMEMGIWECCELLNDVIDESDPDLDEPQIEHLLQTA
GdMIOX07     YRTNHINQTSDFVKRMRTEYGKLNKMEMGIWECCELLNDVIDESDPDLDEPQIEHLLQTA
GtMIOX07     YRTNHINQTSDFVKRMRTEYGKLNKMEMGIWECCELLNDVIDESDPDLDEPQIEHLLQTA
GmMIOX07     YRTNHINQTSDFVKRMRTEYGKLNKMEMGIWECCELLNDVIDESDPDLDEPQIEHLLQTA
GherMIOX02   YRTNHINQTYDFVKRMRTEYGKLNKMEMGIWECCELLNDVIDESDPDLDEPQIEHLLQTA
GkkMIOX02    ...............MRTEYGKLNKMEMGIWECCELLNDVIDESDPDLDEPQIEHLLQTA
GdMIOX02     ...............MRTEYGKLNKMEMGIWECCELLNDVIDESDPDLDEPQIEHLLQTA
ATMIOX01     YRVNHIGQTVDFVRKMREEYEKLNRTEMSIWECCELLNEFIDESDPDLDEPQIEHLLQTA
GaMIOX06     YRTNHINQTVDFVRRMREECGKMERVEMSLWECCELLNEYVDESDPDLDEPQIQHLLQTA
GhMIOX05     YRTNHINQTVDFVRRMREECGKMERVEMSLWECCELLNEYVDESDPDLDEPQIQHLLQTA
GbMIOX11     ......................MERVEMSIWECCELLNEYVDESDPDLDEPQIQHLLQTA
GdMIOX10     YRTNHINQTVEQVRRMRQECGKMERVEMSIWECCELLNEYVDESDPDLDEPQIQHLLQTA
GtMIOX10     YRTNHINQTLRQVRRMRQECGKMERVEMSIWECCELLNEYVDESDPDLDEPQIQHLLQTA
GrMIOX02     YRTNHINQTVDFVRRMRQECGKMERVEMSIWECCELLNEYVDESDPDLDEPQIQHLLQTA
GhMIOX11     YRTNHINQTVRRMR...QECGKMERVEMSIWECCELLNEYVDESDPDLDEPQIQHLLQTA
GmMIOX10     YRTNHINQTVRRMR...QECGKMERVEMSIWECCELLNEYVDESDPDLDEPQIQHLLQTA
GbMIOX05     ......................MERVEMSLWECCELLNEYVDESDPDLDEPQIQHLLQTA
GherMIOX05   YRTNHINQTVDFVRRMREECGKMERVEMSLWECCELLNEYVDESDPDLDEPQIQHLLQTA
GkkMIOX06    ......................MERVEMSIWECCELLNEYVDESDPDLDEPQIQHLLQTA
```

```
              120       130       140       150       160       170
GdMIOX01      EAIRKDYPNEDWYDN.FAFTDLGKILVLPKFGGLPQWAVVGDTFPVGCAFDESNVHHKYF
GtMIOX01      EAIRKDYPNEDWYDN.FAFTDLGKILVLPKFGGLPQWAVVGDTFPVGCAFDQSNVHHKYF
GbMIOX01      EAIRKDYPNEDWYDN.FAFTDLGKILVLPKFGGLPQWTVVGDTFPVGCAFDESNVHHKYF
GaMIOX01      EAIRKDYPNEDWLHLTALIHDLGKILVLPKFGGLPQWAVVGDTFPVGCAFDESNVHHKYF
GkkMIOX01     EAIRKDYPNEDWLHLTALIHDLGKILVLPKFGGLPQWAVVGDTFPVGCAFDESNVHHKYF
GhMIOX01      EAIRKDYPNEDWLHLTALIHDLGKILVLPKFGGLPQWAVVGDTFPVGCAFDESNVHHKYF
GmMIOX01      EAIRKDYPNEDWLHLTALIHDLGKILVLPKFGGLPQWAVVGDTFPVGCAFDESNVHHKYF
GherMIOX01    EAIRKDYPNEDWLHLTALIHDLGKILVLPKFGGLPQWAVVGDTFPVGCAFDESNVHHKYF
GbMIOX07      EAIRKDYPNEDWLHLTALIHDLGKILVLPKFGGLPQWAVVGDTFPVGCAFDESNVHHKYF
GtMIOX06      EAIRKDYPNEDWLHLTALIHDLGKILVLPKFGGLPQWAVVGDTFPVGCAFDESNVHHKYF
GrMIOX01      EAIRKDYPNENWLHLTALIHDLGKILVLPKFGGLPQWAVVGDTFPVGCAFDESNVHHKYF
GhMIOX07      EAIRKDYPNEDWLHLTALIHDLGKILVLPKFGGLPQWAVVGDTFPVGCAFDESNVHHKYF
GdMIOX06      EAIRKDYPNEDWLHLTALIHDLGKILVLPKFGGLPQWAVVGDTFPVGCAFDESNVHHKYF
GmMIOX06      EAIRKDYPNEDWLHLTALIHDLGKILVLPKFGGLPQWAVVGDTFPVGCAFDESNVHHKYF
GmMIOX05      EAIEKTILMK.........TDLGKILVLPKFGELPQWAVVGDTFPVGCAFDEFNVHYKYF
GtMIOX05      EAIEKTILMK.........TDLGKILVLPKFGELPQWAVVGDTFPY.............F
GdMIOX05      EAIEKTILMK.........TDLGKILVLPKFGELPQWAVVGDTFPVGCAFDEFNVHYKYF
GaMIOX05      EAIRKDYPNEDWLHLTALIHDLGKILVLPKFGELPQWAVVGDTFPVGCAFDEFNVHYKYF
GbMIOX06      DWLHLTALIH..........DLGKILVLPKFGELPQWAVVGDTFPVGCAFDEFNVHYKYF
GhMIOX06      DWLHLTAHIH..........DLGKILVLPKFGELPQWAVVGDTFPVGCAFDEFNVHYKYF
GhMIOX12      EAIRKDYPNEDWLHLTALIHDLGKILVLPKFGELPQWAVVGDTFPVGCAFDEFNVHYKYF
GtMIOX11      EAIRKDYPNEDWLHLTALIHDLGKILVLPKFGELPQWAVVGDTFPVGCAFDEFNVHYKYF
GbMIOX12      EAIRKDYPNEDWLHLTALIHDLGKILVLPKFGELPQWAVVGDTFPVGCAFDEFNVHYKYF
GrMIOX03      EAIRKDYPNEDWLHLTALIHDLGKILVLPKFGELPQWAVVGDTFPVGCAFDEFNVHYKYF
GherMIOX06    EAIRKDYPNEDWLHLTALIHDLGKILVLPKFGELPQWAVVGDTFPVGCAFDEFNVHYKYF
GdMIOX11      EAIRKDYPNEDWLHLTALIHDLGKILVLPKFGELPQWAVVGDTFPVGCAFDEFNVHYKYF
GmMIOX11      EAIRKDYPNEDWLHLTALIHDLGKILVLPKFGELPQWAVVGDTFPVGCAFDEFNVHYKYF
GkkMIOX05     EAIRKDYPNEDWLHLTALIHDLGKILVLPNFGELPQWAVVGDTFPVGCAYDEFNVHYKCF
ATMIOX03      EAIRKDYPNEDWLHLTALIHDLGKVITLPQFGGLPQWAVVGDTFPVGCAFDESNVHHKYF
ATMIOX04      EAIRKDYPNEDWLHLTALIHDLGKVLTLPQFGGLPQWAVVGDTFPVGCAFDESNVHHKYF
GaMIOX03      EAIRKDYPNEDWLHLTALIHDLGKVLLLPKFGGLPQWAVVGDTFPLGCAFDEANIHHRYF
GdMIOX03      EAIRKDYPNEDWLHLTALIHDLGKVLLLPKFGGLPQWAVVGDTFPLGCAFDEANIHHRYF
GherMIOX03    EAIRKDYPNEDWLHLTALIHDLGKVLLLPKFGGLPQWAVVGDTFPLGCAFDEANIHHRYF
GbMIOX03      EAIRKDYPNEDWLHLTALIHDLGKVLLLPKFGGLPQWAVVGDTFPLGCAFDEANIHHRYF
GtMIOX03      EAIRKDYPNEDWLHLTALIHDLGKVLLLPKFGGLPQWAVVGDTFPLGCAFDEANIHHRYF
GhMIOX03      EAIRKDYPNEDWLHLTALIHDLGKVLLLPKFGGLPQWAVVGDTFPLGCAFDEANIHHRYF
GmMIOX03      EAIRKDYPNEDWLHLTALIHDLGKVLLLPKFGGLPQWAVVGDTFPLGCAFDEANIHHRYF
GkkMIOX03     EAIRKDYPNEDWLHLTALIHDLGKVLLLPKFGGLPQWAVVGDTFPLGCAFDEANIHHKYF
GtMIOX08      EAIRKDYPNEDWLHLTALIHDLGKVLLLPKFGGLPQWAVVGDTFPLGCAFDEANIHHKYF
GbMIOX09      EAIRKDYPNEDWLHLTALIHDLGKVLLLPKFGGLPQWAVVGDTFPLGCAFDEANIHHKYF
GdMIOX08      EAIRKDYPNEDWLHLTALIHDLGKVLLLPKFGGLPQWAVVGDTFPLGCAFDEANIHHKYF
GhMIOX09      EAIRKDYPNEDWLHLTALIHDLGKVLLLPKFGGLPQWAVVGDTFPLGCAFDEANIHHKYF
GrMIOX05      EAVRKDYPNEDWLHLTALIHDLGKVLLLPKFGGLPQWAVVGDTFPLGCAFDEANIHHKYF
GmMIOX08      STSCSRYPNEDWLHLTALIHDLGKVLLLPKFGGLPQWAVVGDTFPLGCAFDEANIHHKYF
ATMIOX02      EAIRRDYPDEDWLHLTALIHDLGKVLLLPEFGGLPQWAVVGDTFPVGCTFDSANIHHKYF
GherMIOX04    EAIRKDYPDEDWLHLTGLIHDLGKVLLHPSFGGLPQWAVVGDTYPVGCAFDKSIVHHKYF
GhMIOX04      EAIRKDYPDEDWLHLTGLIHDLGKVLLHPSFGGLPQWAVVGDTYPVGCAFDKSIVHHKYF
GaMIOX04      EAIRKDYPDEDWLHLTGLIHDLGKVLLHPSFGGLPQWAVVGDTYPVGCAFDKSIVHHKYF
GbMIOX04      EAIRKDYPDEDWLHLTGLIHDLGKVLLHPSFGGLPQWAVVGDTYPVGCAFDKSIVHHKYF
GtMIOX04      EAIRKDYPDEDWLHLTGLIHDLGKVLLHPSFGGLPQWAVVGDTYPVGCAFDKSIVHHKYF
GmMIOX04      EAIRKDYPDEDWLHLTGLIHDLGKVLLHPSFGGLPQWAVVGDTYPVGCAFDKSIVHHKYF
GdMIOX09      EAIRKDYPDEDWLHLTGLIHDLGKVLLHPSFGGLPQWAVVGDTYPVGCAFDKSIVHHKYF
GtMIOX09      EAIRKDYPDEDWLHLTGLIHDLGKVLLHPSFGGLPQWAVVGDTYPVGCAFDKSIVHHKYF
GbMIOX10      EAIRKDYPDEDWLHLTGLIHDLGKVLLHPSFGGLPQWAVVGDTYPVGCAFDKSIVHHKYF
GhMIOX10      EAIRKDYPDEDWLHLTGLIHDLGKVLLHPSFGGLPQWAVVGDTYPVGCAFDKSIVHHKYF
GmMIOX09      EAIRKDYPDEDWLHLTGLIHDLGKVLLHPSFGGLPQWAVVGDTYPVGCAFDKSIVHHKYF
GrMIOX06      EAIRKDYPDEDWLHLTGLIHDLGKVLLHPSFGGLPQWAVVGDTYPVGCAFDKSIVHHKYF
GdMIOX04      EAIRKDYPDEDWLHLTGLIHDLGKVLLHPSFGGLPQWAVVGDTYPAGCAFDKSIVHHKYF
GkkMIOX04     EAIRKDYPDEDWLHLTGLIHDLGKVLLHPSFGGLPQWAVVGDTYPVGCAFDKSIVHHKYF
GhMIOX02      EAIRKDYPNEDWMHLTGLIHDLGKVLLHPGFGELPQWAVVGDTFPVGCAFDKSIVHHKYF
GtMIOX02      EAIRKDYPNEDWMHLTGLIHDLGKVLLHPGFGELPQWAVVGDTFPVGCAFDKSIVHHKYF
GbMIOX02      EAIRKDYPNEDWMHLTGLIHDLGKVLLHPGFGELPQWAVVGDTFPVGCAFDKSIVHHKYF
GmMIOX02      EAIRKDYPNEDWMHLTGLIHDLGKVLLHPGFGELPQWAVVGDTFPVGCAFDKSIVHHKYF
GaMIOX02      EAIRKDYPNEDWMHLTGLIHDLGKVLLHPGFG.....SFHSDTFPVGCAFDKSIVHHKYF
GhMIOX08      EAIRKDYPNEDWMHLTGLIHDLGKVLLHPGFGELPQWAVVGDTFPVGCAFDKSIVHHKKS
GrMIOX04      EAIRKDYPNEDWMHLTGLIHDLGKVLLHPGFGELPQWAVVGDTFPVGCAFDKSIVHHKYF
GbMIOX08      EAIRKDYPNEDWMHLTGLIHDLGKVLLHPGFGELPQWAVVGDTFPVGCAFDKSIVHHKYF
GdMIOX07      EAIRKDYPNEDWMHLTGLIHDLGKVLLHPGFGELPQWAVVGDTFPVGCAFDKSIVHHKYF
GtMIOX07      EAIRKDYPNEDWMHLTGLIHDLGKVLLHPGFGELPQWAVVGDTFPIGCAFDKSIVHYKYF
GmMIOX07      EAIRKDYPNEDWMHLTGLIHDLGKVLLHPGFGELPQWAVVGDTYPVGCAFDKSIVHHKYF
GherMIOX02    EAIRKDYPNEDWMHLTGLIHDLGKVLLHPGFGELPQWAVY...................F
GkkMIOX02     EAIRKDYPNEDWLHLTGLIHDLGKVLLHPGFGELPQWAVVGDTFPVGCAFDKSIVHHKYF
GdMIOX02      EAIRKDYPNEDWMHLTGLIHDLGKVLLHPGFGELPQWAVVGDTFPVGCAFDKSIVHHKYF
ATMIOX01      EAIRKDYPDEDWLHLTGLIHDLGKVLLHSSFGELPQWAVVGDTFPVGCAFDESIVHHKYF
GaMIOX06      EAIRKDYPDEDWLHLTALIHDLGKVLLHPAFGELPQWAMVGDTFPVGCAFDESIVDHKYF
GhMIOX05      EAIRKDYPDEDWLHLTALIHDLGKVLLHPAFGELPQWAMVGDTFPVGCAFDESIVDHKVI
GbMIOX11      EAIRKDYPDEDWLHLTALIHDLGKVLLHPAFGELPQWAMVGDTFPVGCAFDESIVDHKEV
GdMIOX10      EAIRKDYPDEDWLHLTALIHDLGKVLLHPAFGELPQWAMVGDTFPVGCAFDESIVDHKYF
GtMIOX10      EAIRKDYPDEDWLHLTALIHDLGKVLLHPAFGELPQWAMGGDTFPVGCAFDESIVDHKYF
GrMIOX02      EAIRKDYPDEDWLHLTALIHDLGKVLLHPAFGELPQWAMVGDTFPVGCAFDESIVDHKYF
GhMIOX11      EAIRKDYPDEDWLHLTALIHDLGKVLLHPAFGELPQWAMVGDTFPVGCAFDESIVDHK..
GmMIOX10      EAIRKDYPDEDWLHLTALIHDLGKVLLHPAFGELPQWAMVGDTFPVGCAFDESIVDHK..
GbMIOX05      EAIRKDYPDEDWLHLTALIHDLGKVLLHPAFGELPQWAMVGDTFPVGCAFGESIVDHKYF
GherMIOX05    EAIRKDYPDEDWLHLTALIHDLGKVLLHPAFGELPQWAMVGDTFPVGCAFDESIVDHKYF
GkkMIOX06     EAIRKDYPDEDWLHLTALIHDLGKVLLHPAFGELPQWAVVGDTFPVGCAFDESIVHHKYF
```

```
              180       190       200       210       220
GdMIOX01      KENPDFNNPKYNTRNGVYIEGCGLDNVLMSWGHDDYMYMVAKENGT..............
GtMIOX01      KENPDFNNPKYNTRNGVYIEGCGLDNVLMSWGHDDYMYMADKENGT..............
GbMIOX01      KENPDFNNPKYNTRNGVYIEGCGLDNVLMSWGHDDYMYMVAKENGT..............
GaMIOX01      KENPDFNNPKYNTRNGVYIEGCGLDNVLMSWGHDDYMYMVAKENGT..............
GkkMIOX01     KGNPDFNNPKYNTRNGVYMEGCGLDNVLMSWGHDDYMYMVAKENGT..............
GhMIOX01      KENPDFNNPKYNTRNGVYIEGCGLDNVLMSWGHDDYMYMVAKENGT..............
GmMIOX01      KENPDFNNPKYNTRNGVYIEGCGLDNVLMSWGHDDYMYMVAKENGT..............
GherMIOX01    KENPDFNNPKYNTRNGVYIEGCGLDNVLMSWGHDDYMYMVAKENGT..............
GbMIOX07      KENPDFNNPKHNTRNGVYIEGCGLDNVLMSWGHDDYMYMVAKENGT..............
GtMIOX06      KENPDFNNPKHNTRNGVYIEGCGLDNVLMSWGHDDYMYMVAKENGT..............
GrMIOX01      KENPDFNNPKYNTRNGVYIEGCGLDNVLMSWGHDDYMYMVAKENGT..............
GhMIOX07      KENPDFNNPKHNTRNGVYIEGCGLDNVLMSWGHDDYMYMVAKENGT..............
GdMIOX06      KENPDFNNPKHNTRNGVYIEGCGLDNVLMSWGHDDYMYMVAKENGT..............
GmMIOX06      KENPDFNNPKHNTRNGVYIEGCGLDNVLMSWGHDDYMYMVAKENGT..............
GmMIOX05      KENPDFKNPKYNTNNGVYSEGCGLDNLLMSWGHDDYMYMVAKENGT..............
GtMIOX05      KENPDFKNPKYNTNNGVYSEGCGLDNLLMSWGHDDYMYMVAKENGT..............
GdMIOX05      KENPDFKNPKYNTNNGVYSEGCGLDNLLMSWGHDDYMYMVAKENGT..............
GaMIOX05      KENPDFKNPKYNTNNGVYSEGCGLDNLLMSWGHDDYMYMVAKENGT..............
GbMIOX06      KENPDFKNPKYNTNNGVYSEGCGLDNLLMSWGHDDYMYMVAKENGT..............
GhMIOX06      KENPDFKNPKYNTNNGVYSEGCGLDNLLMSWGHDDYMYMVAKENGT..............
GhMIOX12      RENPDFKNPKYNTNNGVYSEGCGLDNLLMSWGHDDYMYMVAKENGT..............
GtMIOX11      RENPDFKNPKYNTNNGVYSEGCGLDNLLMSWGHDDYMYMVAKENGT..............
GbMIOX12      RENPDFKNPKYNTNNGVYSEGCGLDNLLMSWGHDDYMYMVAKENGT..............
GrMIOX03      RENPDFKNPKYNTNNGVYSEGCGLDNLLMSWGHDDYMYMVAKENGT..............
GherMIOX06    KENPDFKNPKYNTNNGVYSEGCGLDNLLMSWGHDDYMYMVAKENGT..............
GdMIOX11      RENPDFKNPKYNTNNGVYSEGCGLDNLLMSWGHDDYMYMVAKENGT..............
GmMIOX11      RENPDFKNPKYNTNNGVYSEGCGLDNLLMSWGHDDYMYMVAKENGT..............
GkkMIOX05     KENPDFKNTKYNTNNGVYSEGCGLDNLLMSW........VAKENGT..............
ATMIOX03      VENPDFHNETYNTKNGIYSEGCGLNNVMMSWGHDDYMYLVAKENGS..............
ATMIOX04      MENPDFNNPKYNTKAGIYSEGCGLENVLMSWGHDDYMYLVAKENGS..............
GaMIOX03      KENPDCNNPSYNTKNGIYWDGCGLDNVTISWGHDDYMYLVAKENGT..............
GdMIOX03      KENPDYNNPSYNTKNGIYWDGCGLDNVTISWGHDDYMYLVAKENGT..............
GherMIOX03    KENPDYNNPSYNTKNGIYWDGCGLDNVTISWGHDDYMYLVAKENGT..............
GbMIOX03      KENPDYNNPSYNTKNGIYWDGCGLDNVTISWGHDDYMYLVAKENGT..............
GtMIOX03      KENPDYNNPSYNTKNGIYWDGCGLDNVTISWGHDDYMYLVAKENGT..............
GhMIOX03      KENPDYNNPSYNTKNGIYWDGCGLDNVTISWGHDDYMYLVAKENGT..............
GmMIOX03      KENPDYNNPSYNTKNGIYWDGCGLDNVTISWGHDDYMYLVAKENGT..............
GkkMIOX03     KENPDYNNPSYNTRNGIYSDGCGLDNVTISWGHDDYMYLVAKENIT..............
GtMIOX08      KENPDYNNPSYNTKNGIYWDGCGLDNVTISWGHDDYMYLVAKENGT..............
GbMIOX09      KENPDYNNPSYNTKNGIYWDGCGLDNVTISWGHDDYMYLVVKENGT..............
GdMIOX08      KENPDYNNPSYNTKNGIYWDGCGLDNVTISWGHDDYMYLVVKENGT..............
GhMIOX09      KENPDYNNPSYNTKNGIYWDGCGLENVTISWGHDDYMYLVAKENGT..............
GrMIOX05      KENPDYNNPSYNTKNGIYWDGCGLDNVTISWGHDDYMYLVAKENGT..............
GmMIOX08      KENPDYNNPSYNTKNGIYWDGCGLDNVTISWGHDDYMYLVAKENGT..............
ATMIOX02      KGNHDINNPKYNTKNGVYTEGCGLDNVLMSWGHDDYMYLVAKKNGT..............
GherMIOX04    EENPDYHNPAYNTKYGVYSEGCGLNNVMMSWGHDDYMYLVAKENNT..............
GhMIOX04      EENPDYHNPAYNTKYGVYSEGCGLNSVMMSWGHDDYMYLVAKENNT..............
GaMIOX04      EENPDYHNPAYNTKYGVYSEGCGLNNVMMSWGHDDYMYLVAKENNT..............
GbMIOX04      EENPDYHNPAYNTKYGVYSEGCGLNNVMMSWGHDDYMYLVAKENNT..............
GtMIOX04      EENPDYHNPAYNTKYGVYSEGCGLNNVMMSWGHDDYMYLVAKENNT..............
GmMIOX04      EENPDYHNPAYNTKYGVYSEGCGLNNVMMSWGHDDYMYLVAKENNT..............
GdMIOX09      EENPDYHNPAYNTKYGVYSEGCGLNNVMMSWGHDDYMYLVAKENDT..............
GtMIOX09      EENPDYHNPAYNTKYGVYSEGCGLNNVMMSWGHDDYMYLVAKENDT..............
GbMIOX10      EENPDYHNPAYNTKYGVYSEGCGLNNVMMSWGHDDYMYLVAKENDT..............
GhMIOX10      EENPDFHNPAYNTKYGVYSEGCGLNNVMMSWGHDDYMYLVAKENDT..............
GmMIOX09      EENPDYHNPAYNTKYGVYSEGCGLNNVMMSWGHDDYMYLVAKENKT..............
GrMIOX06      EENPDYHNPAYNTKYGVYSEGCGLNNVMMSWGHDDYMYLVAKENKT..............
GdMIOX04      EENPDYHNPAYNTKYGVYSEGCGLNNVMMSWGHDDYMYLVAKENNT..............
GkkMIOX04     EENPDYHNPAYNTKYGVYSEGCGLNNVLMSWGHDDYMYLVAKENNT..............
GhMIOX02      EENPDYHNSDYNTKYGVYSEGCGLNNVMMSWGHDDYMYLVSKGNNT..............
GtMIOX02      EENPDYHNSDYNTKYGVYSEGCGLNNVMMSWGHDDYMYLVSKGNNT..............
GbMIOX02      EENPDYHNSDYNTKYGVYSEGCGLNNVMMSWGHDDYMYLVSKGNNT..............
GmMIOX02      EENPDYHNSDYNTKYGVYSEGCGLNNVMMSWGHDDYMYLVSKGNNT..............
GaMIOX02      EENPDYHNSDYNTKYGVYSEGCGLNNVMMSWGHDDYMYLVAKGNNT..............
GhMIOX08      YSFSSVGEQR..................................................
GrMIOX04      EENPDYHNSDYNTKYGVYSEGCGLNNVMMSWGHDDYMYLVAKGNNT..............
GbMIOX08      EENPDYHNSDYNTKYGVYSEGCGLNNVMMSWGHDDYMYLVAKGNNT..............
GdMIOX07      EENPDYHNSDYNTKYGVYSEGCGLNNVMMSWGHDDYMYLVAKGNNT..............
GtMIOX07      EENPDYHNSDYNTKYGVYSEGCGLNNVMMLWGHDDYMYLVSKGNNT..............
GmMIOX07      EENPDYHNSDYNTKYGVYSEGCGLNNVMMSWGHDDYMYLVAKGNNT..............
GherMIOX02    EENLDYHNSDYNTKYGVYSEGCGLNNVMMSWGHDDYMYLVAKGNNT..............
GkkMIOX02     EENPDYHNSDYNTKYGVYSEGCGLNNVMMSWGHDDYMYLVAKGNNT..............
GdMIOX02      EENPDYHNSDYNTKYGVYSEGCGLNNVMMSWGHDDYMYLVSKGNNT..............
ATMIOX01      KENPDYDNPSYNSKYGIYTEGCGLDNVLMSWGHDDYMYLVAKENQT..............
GaMIOX06      QENLDNKNPAYNTKYGIYSEGFGLHNVLMSWGHDDYMYLLHFQPHFSFFFCFKLNFIQHH
GhMIOX05      TH...............FYTCFGLHNVLMSWGHDDYMYLLHFQPHFSFFFCFNLNFIQHY
GbMIOX11      TKS.........................................................
GdMIOX10      QENPDNKNPAYNTKYGIYSEGCGLHNVLMSWGHDDYMYLF...RCG..............
GtMIOX10      QENPDNKNPAYNTKYGIYSEGCGLHNVLMSWGHDDYMYLF...RCG..............
GrMIOX02      QENPDNKNPAYNTKYGIYSEGCGLHNVLMSWGHDDYMYLV...HFQPHFSFFIF......
GhMIOX11      .ENPDNKNPAYNTKYGIYSEGCGLHNVLMSWGHVDYMYLSNIS.................
GmMIOX10      .ENPDNKNPAYNTKYGIYSEGCGLHNVLMSWGHDDYMCG.....................
GbMIOX05      QENLDNKNPAYNNLL.............................................
GherMIOX05    QENLDNKNPAYNTKYGIYSEGFGLHNVLMSWGHDDYMYLLHFQPHFSFFFCFKLNFIQHH
GkkMIOX06     QENPDNKNPAYNTKYGIYSEGCGLHNVWMSWGHDDYMYLVVNENKSTLLSPGLFIIRYHS
```

```
                          230
GdMIOX01     ........TLPSAGLFIVRYHSFY....................................
GtMIOX01     ........TLPSAGLFIVRYHSFY....................................
GbMIOX01     ........TLPSAGLFIVRYHSFY....................................
GaMIOX01     ........TLPSAGLFIVRYHSFY....................................
GkkMIOX01    ........TLPSAGLFIIRYHSFY....................................
GhMIOX01     ........TLPSAGLFIVRYHSFY....................................
GmMIOX01     ........TLPSAGLFIVRYHSFY....................................
GherMIOX01   ........TLPSAGLFIVRYHSFY....................................
GbMIOX07     ........TLPSAGLFIVRYHSFY....................................
GtMIOX06     ........TLPSAGLFIVRYHSFY....................................
GrMIOX01     ........TLPSAGLFIVRYHSFY....................................
GhMIOX07     ........TLPSAGLFIVRYHSFY....................................
GdMIOX06     ........TLPSAGLFIVRYHSFYRKHFFPYRCCFVSKYHNPFYLISTYLIA........
GmMIOX06     ........TLPSAGLFIVRYHSFY....................................
GmMIOX05     ........TLPQAGLFIIRYHSFYP...................................
GtMIOX05     ........TLPQAGLFIIRYHSFYP...................................
GdMIOX05     ........TLPQAGLFIIRYHSFYP...................................
GaMIOX05     ........TLPQAGLFIIRYHSFYP...................................
GbMIOX06     ........TLPQAGLFIIRYHSFYP...................................
GhMIOX06     ........TLPQAGLFIIRYHSFYP...................................
GhMIOX12     ........TLPQAGLFIIRYHSFYP...................................
GtMIOX11     ........TLPQAGLFIIRYHSFYP...................................
GbMIOX12     ........TLPQAGLFIIRYHSFYP...................................
GrMIOX03     ........TLPQAGLFIIRYHSFYP...................................
GherMIOX06   ........TLPQAGLFIIRYHSFYP...................................
GdMIOX11     ........TLPQAGLFIIRYHSFYP...................................
GmMIOX11     ........TLPQAGLFIIRYHSFYP...................................
GkkMIOX05    ........TLPQAGLFIIRYHSFYS...................................
ATMIOX03     ........TLPSAGQFIIRYHSFYP...................................
ATMIOX04     ........TLPSPGLFIIRYHSFYP...................................
GaMIOX03     ........TLPSAGLFIIRYHSLYP...................................
GdMIOX03     ........TLPSAGLFIIRYHSLYP...................................
GherMIOX03   ........TLPSAGLFIIRYHSLYP...................................
GbMIOX03     ........TLPSAGLFIIRYHSLYP...................................
GtMIOX03     ........TLPSAGLFIIRYHSLYP...................................
GhMIOX03     ........TLPSAGLFIIRYHSLYP...................................
GmMIOX03     ........TLPSAGLFIIRYHSLYP...................................
GkkMIOX03    ........TLPSAG..............................................
GtMIOX08     ........TLPSAGLFIIRYHSLYP...................................
GbMIOX09     ........TLPSAGLFIIRYHSLYP...................................
GdMIOX08     ........TLPSAGLFIIRYHSLYP...................................
GhMIOX09     ........TLPSAGLFIIRYHSLYP...................................
GrMIOX05     ........TLPSAGLFIIRYHSLYP...................................
GmMIOX08     ........TLPSAGLFIIRYHSLYP...................................
ATMIOX02     ........TLPHAGLFIIRYHSFYP...................................
GherMIOX04   ........TLPSAALFIIRYHSFYA...................................
GhMIOX04     ........TLPSAALFIIRYHSFYA...................................
GaMIOX04     ........TLPSAALFIIRYHSFYA...................................
GbMIOX04     ........TLPSAALFIIRYHSFYA...................................
GtMIOX04     ........TLPSAALFIIRYHSFYA...................................
GmMIOX04     ........TLPSAALFIIRYHSFYA...................................
GdMIOX09     ........TLPSAALFIIRYHSFYA...................................
GtMIOX09     ........TLPSAALFIIRYHSFYA...................................
GbMIOX10     ........TLPSAALFIIRYHSFYA...................................
GhMIOX10     ........TLPSAALFIIRYHSFYA...................................
GmMIOX09     ........TLPSAALFIIRCHSFYA...................................
GrMIOX06     ........TLPSAALFIIRYHSFYA...................................
GdMIOX04     ........TLPSAALFIIRYHSFYA...................................
GkkMIOX04    ........TLPPAALFIIRYHSFYA...................................
GhMIOX02     ........TLPPAALFIIRYHSFYA...................................
GtMIOX02     ........TLPPAALFIIRYHSFYA...................................
GbMIOX02     ........TLPPAALFIIRYHSFYA...................................
GmMIOX02     ........TLPPAALFIIRYHSFYA...................................
GaMIOX02     ........TLPPAALFIIRYHSFYA...................................
GhMIOX08     ............................................................
GrMIOX04     ........TLPPAALFIIRYHSFYA...................................
GbMIOX08     ........TLPPAALFIIRYHSFYA...................................
GdMIOX07     ........TLPPAALFIIRYHSFYA...................................
GtMIOX07     ........TLPPAALFIIRYHSFYA...................................
GmMIOX07     ........TLPPAALFIIRYHSFYA...................................
GherMIOX02   ........TLPPAALFIIRYHSFYA...................................
GkkMIOX02    ........TLPPAALFIIRYHSFYA...................................
GdMIOX02     ........TLPPAALFIIRYHSFYA...................................
ATMIOX01     ........TLPSAGLFIIRYHSFYA...................................
GaMIOX06     MVVNEDKSTFSSLGLFIIRYHSFYA...................................
GhMIOX05     MVVKEDKSTFPSLGLFIIRYHSFYA...................................
GbMIOX11     ............................................................
GdMIOX10     ............................................................
GtMIOX10     ............................................................
GrMIOX02     .................LYKSXIXL...................................
GhMIOX11     .......................IL...................................
GmMIOX10     ............................................................
GbMIOX05     ............................................................
GherMIOX05   MVVNEDKSTFPSLGLFIIRYHSFYVNMIFTAKAKFTYMLRKSSPTIYLSSTRKLPSHSIR
GkkMIOX06    ............................................................
```

```
GdMIOX01     ............................................................
GtMIOX01     ............................................................
GbMIOX01     ............................................................
GaMIOX01     ............................................................
GkkMIOX01    ............................................................
GhMIOX01     ............................................................
GmMIOX01     ............................................................
GherMIOX01   ............................................................
GbMIOX07     ............................................................
GtMIOX06     ............................................................
GrMIOX01     ............................................................
GhMIOX07     ............................................................
GdMIOX06     ............................................................
GmMIOX06     ............................................................
GmMIOX05     ............................................................
GtMIOX05     ............................................................
GdMIOX05     ............................................................
GaMIOX05     ............................................................
GbMIOX06     ............................................................
GhMIOX06     ............................................................
GhMIOX12     ............................................................
GtMIOX11     ............................................................
GbMIOX12     ............................................................
GrMIOX03     ............................................................
GherMIOX06   ............................................................
GdMIOX11     ............................................................
GmMIOX11     ............................................................
GkkMIOX05    ............................................................
ATMIOX03     ............................................................
ATMIOX04     ............................................................
GaMIOX03     ............................................................
GdMIOX03     ............................................................
GherMIOX03   ............................................................
GbMIOX03     ............................................................
GtMIOX03     ............................................................
GhMIOX03     ............................................................
GmMIOX03     ............................................................
GkkMIOX03    ............................................................
GtMIOX08     ............................................................
GbMIOX09     ............................................................
GdMIOX08     ............................................................
GhMIOX09     ............................................................
GrMIOX05     ............................................................
GmMIOX08     ............................................................
ATMIOX02     ............................................................
GherMIOX04   ............................................................
GhMIOX04     ............................................................
GaMIOX04     ............................................................
GbMIOX04     ............................................................
GtMIOX04     ............................................................
GmMIOX04     ............................................................
GdMIOX09     ............................................................
GtMIOX09     ............................................................
GbMIOX10     ............................................................
GhMIOX10     ............................................................
GmMIOX09     ............................................................
GrMIOX06     ............................................................
GdMIOX04     ............................................................
GkkMIOX04    ............................................................
GhMIOX02     ............................................................
GtMIOX02     ............................................................
GbMIOX02     ............................................................
GmMIOX02     ............................................................
GaMIOX02     ............................................................
GhMIOX08     ............................................................
GrMIOX04     ............................................................
GbMIOX08     ............................................................
GdMIOX07     ............................................................
GtMIOX07     ............................................................
GmMIOX07     ............................................................
GherMIOX02   ............................................................
GkkMIOX02    ............................................................
GdMIOX02     ............................................................
ATMIOX01     ............................................................
GaMIOX06     ............................................................
GhMIOX05     ............................................................
GbMIOX11     ............................................................
GdMIOX10     ............................................................
GtMIOX10     ............................................................
GrMIOX02     ............................................................
GhMIOX11     ............................................................
GmMIOX10     ............................................................
GbMIOX05     ............................................................
GherMIOX05   NILTLKSIRNFYVLNTGAKIPAIGLGTWQSGGDLCVDAVKTAFSVGYRHIDCAHLYGNEI
GkkMIOX06    ............................................................
```

```
               240       250       260       270       280       290
GdMIOX01       ..PLHKYGAYSQFMNEEDKENFKWLKVFNKYDLYSKSKLLVDVEKVKPYYESLIAKYFPA
GtMIOX01       ..PLHKYGAYSQFMNEEDKENFKWLKVFNKYDLYSKSKLLVDVEKVKPYYESLIAKYFPA
GbMIOX01       ..PLHKYGAYSQFMNEEDKENFKWLKVFNKYDLYSKSKLLVDVEKVKPYYESLIAKYFPA
GaMIOX01       ..PLHKYGAYSQFMNEEDKENFKWLKVFNKYDLYSKSKLLVDVEKVKPYYESLIAKYFPA
GkkMIOX01      ..PLHKYGAYSQFMNEEDKENFKWLKVFK...............................
GhMIOX01       ..PLHKYGAYSQFMNEEDKENFKWLKVFNKYDLYSKSKLLVDVEKVKPYYESLIAKYFPA
GmMIOX01       ..PLHKYGAYSQFMNEEDKENFKWLKVFNKYDLYSKSKLLVDVEKVKPYYESLIAKYFPA
GherMIOX01     ..PLHKYGAYSQFMNEEDKENFKWLKVFNKYDLYSKSKLLVDVEKVKPYYESLIAKYFPA
GbMIOX07       ..PLHKYGAYSQFMKEEDKENFKWLKVFK...............................
GtMIOX06       ..PLHKYGAYSQFMKEEDKENFKWLKVFNKYDLYSKSKVLVDVEKVKPYYESLIAKYFPA
GrMIOX01       ..PLHKYGAYSQLMNKEDKENFKWLKVFNKYDLYSKSKVLVDVEKVKPYYESLIAKYFPA
GhMIOX07       ..PLHKYGAYSQFMKEEDKENFKWLKVFNKYDLYSKSKVLVDVEKVKPYYESLIAKYFPA
GdMIOX06       ..ALHKYGAYSQFMKEEDKENFKWLKVFK...............................
GmMIOX06       ..PLHKYGAYSQFMNEEDKENFKWLKVFNKYDLYSKSKVLVDVEKVKPYYESLIAKYFPA
GmMIOX05       ...LHKHGAYAQFINEEDKKNMKWLHVFNKYDLYSKSKAHVDVEKVKPYYESLIAKYFPA
GtMIOX05       ...LHKHGAYAQFINEEDKKNMKWLHVFNKYDLYSKSKAHVDVK...PYYESLIAKYFPA
GdMIOX05       ...LHKHGAYAQFINEEDKKNMKWLHVFNKYDLYSKSKAHVDVEKVKPYYESLIAKYFPA
GaMIOX05       ...LHKHGAYAQFINEEDKKNMKWLHVFNKYDLYSKSKAHVDVEKVKPYYESLIAKYFPA
GbMIOX06       ...LHKHGAYAQFINEEDKKNMKWLHVFNKYDLYSKSKAHVDVEKVKPYYESLIAKYFPA
GhMIOX06       ...LHKHGAYAQFINEEDKKNMKWLHVFNKYDLYSKSKAHVDVEKVKPYYESLIAKYFPA
GhMIOX12       ...LHKHGAYAQFMNEEDKKNMKWLHVFNKYDLYSKSKAHVDVEKVKPYYESLIAKYFPA
GtMIOX11       ...LHKHGAYAQFMNEEDKKNMKWLHVFNKYDLYSKSKAHVDVEKVKPYYESLIAKYFPA
GbMIOX12       ...LHKHGAYAQFMNEEDKKNMKWLHVFNKYDLYSKSKAHVAVEKVKPYYESLIAKYFPA
GrMIOX03       ...LHKHGAYAQFMNEEDKKNMKWLHVFNKYDLYSKSKAHVDVEKVKPYYESLIAKYFPA
GherMIOX06     ...LHKHGAYAQFINEEDKKNMKWLHVFNKYDLYSKSKAHVDVEKVKPYYESLIAKYFPA
GdMIOX11       ...LHKHGAYAQFMNEEDKKNMKWLHVFNKYDLYSKSKAHVDVEKVKPYYESLIAKYFPA
GmMIOX11       ...LHKHGAYAQFMNEEDKKNMKWLHVFNKYDLYSKSKAHVDVEKVKPYYESLIAKYFPA
GkkMIOX05      ...LHKHGAYAQFMNEEDKKTNEQEFS.................................
ATMIOX03       ...LHTAGEYTHLMNEEDKENLKWLHVFNKYDLYSKSKVHVDVEKVKPYYMSLIKKYFPE
ATMIOX04       ...LHKAGAYTHLMNEEDKENLKWLHVFNKYDLYSKSKVHVNVEKVKPYYMSLIKKYFPE
GaMIOX03       ...LHKEEAYMQFLNDEDKENLKWLRIFNKYDLYSKSKVAVDVEKVKPYYLSLIEKYFPA
GdMIOX03       ...LHKEEAYMQFLNDEDKENLKWLRIFNKYDLYSKSKVAVDVEKVKPYYLSLIEKYFPA
GherMIOX03     ...LHKEEAYMQFLNDEDKENLKWLRIFNKYDLYSKSKVAVDVEKVKPYYLSLIEKYFPA
GbMIOX03       ...LHKEEAYMQFVNDEDKENLKWLRIFNKYDLYSKSKVAVDVEKVKPYYLSLIEKYFPA
GtMIOX03       ...LHKEEAYVQFLNDEDKENLKWLRIFNKYDLYSKSKVAVDVEKVKPYYLSLIEKYFPA
GhMIOX03       ...LHKEEAYMQFLNDEDKENLKWLRIFNKYDLYSKSKVAVDVEKVKPYYLSLIEKYFPA
GmMIOX03       ...LHKEEAYMQFLNDEDKENLKWLRIFNKYDLYSKSKVAVDVEKVKPYYLSLIEKYFPA
GkkMIOX03      .....LEGAYMQFLSDEDKKNMKWLRVFK...............................
GtMIOX08       ...LHKEEAYMQFVNDEDKENLKWLRIFNKSKVAVDVEKVKPYYLSLIEKYFPAKLKW..
GbMIOX09       ...LHKEEAYMQFVNDEDKENLKWLRIFNKYDLYSKSKVAVDVEKVKPYYLSLIEKYFPA
GdMIOX08       ...LHKEEAYMQFVNDEDKENLKWLRIFNKYDLYSKSKVAVDVEKVKPYYLSLIEKYFPA
GhMIOX09       ...LHKEEAYMQFVNDEDKENLKWLRIFNKYDLYSKSKVAVDVEKVKPYYLSLIEKYFPA
GrMIOX05       ...LHTEEAYMQFLNDEDKENLKWLRIFNKYDLYSKSKVAVDVEKVKPYYLSLIEKYFPA
GmMIOX08       ...LHKEEAYMQFVNDEDKENLKWLRIFNKYDLYSKSKVAVDVEKVKPYYLSLIEKYFPA
ATMIOX02       ...LHKAGAYTHLMNDEDRDDLKWLHVFNKYDLYSKSKVLVDVEQVKPYYISLINKYFPA
GherMIOX04     ...LHRSGSYKQLMNGEDVENLKWLEIFNKYDLYSKSKVRIDVEKVKPYYLSLIEKSLTN
GhMIOX04       ...LHRSGSYKQLMNGEDVENLKWLEIFNKYDLYSKSKVRIDVEKVKPYYLSLIEKYFPA
GaMIOX04       ...LHRSGSYKQLMNGEDVENLKWLEVFNKYDLYSKSKVRIDVEKVKPYYLSLIEKYFPA
GbMIOX04       ...LHRSGSYKQLMNGEDVENLKWLEIFNKYDLYSKSKVRIDVEKVKPYYLSLIEKYFPA
GtMIOX04       ...LHRSGSYKQLMNGEDVENLKWLEIFNKYDLYSKSKVRIDVEKVKPYYLSLIEKYFPA
GmMIOX04       ...LHRSGSYKQLMNGEDVENLKWLEIFNKYDLYSKSKVRIDVEKVKPYYLSLIEKYFPA
GdMIOX09       ...LHRSGSYKQLMNGEDVENLKWLEIFNKYDLYSKSKVRIDVEKVKPYYLSLIEKYFPA
GtMIOX09       ...LHRSGSYKQLMNGEDVENLKWLEIFNKYDLYSKSKVRIDVEKVKPYYLSLIEKYFPA
GbMIOX10       ...LHRSGSYKQLMNGEDVENLKWLEIFNKYDLYSKSKVRIDVEKVKPYYLSLIEKYFPA
GhMIOX10       ...LHRSGSYKQLMNGEDVENLKWLEIFNKYDLYSKSKVRIDVEKVKPYYLSLIEKYFPA
GmMIOX09       ...LHRSGAYKQLMNGEDVENLKWLEIFNKYDLYSKSKVRIDVEKVKPYYLSLIEKYFPA
GrMIOX06       ...LHRSGAYKQLMNGEDVENLKWLEIFNKYDLYSKSKVRIDVEKVKPYYLSLIEKYFPA
GdMIOX04       ...LHRSGSYKQLMNGEDVENLKWLEIFNKYDLYSKSKVRIDVEKVKPYYLSLIEKYFPA
GkkMIOX04      ...LHRSGSYKQLMNEEDVENLKWLEIFNKYDLYSKSKVRIDVEKVKPYYLSLIEKYFPA
GhMIOX02       ...LHKSGAYRHLMNEEDTENMKWLQIFNKYDLYSKSKVRIDVEKVKPYYLSLIEKYFPA
GtMIOX02       ...LHKSGAYRHLMNEEDTENMKWLQIFNKYDLYSKSKVRIDVEKVKPYYLSLIEKYFPA
GbMIOX02       ...LHKSGAYRHLMNEEDTENMKWLQIFNKYDLYSKSKVRIDVEKVKPYYLSLIEKYFPA
GmMIOX02       ...LHKSGAYRHLMNEEDTENMKWLQIFNKYDLYSKSKVRIDVEKVKPYYLSLIEKYFPA
GaMIOX02       ...LHKSGAYRHLMNEEDTENMKWLQIFNKYDLYSKSKVRIDVEKVKPYYLSLIKKYFPA
GhMIOX08       ............................................................
GrMIOX04       ...MHKSGAYRHLMNEEDTENLKWLQIFNKYDLYSKSKVRIDVEKVKPYYLSLIEKYFPA
GbMIOX08       ...LHKSGAYRHLMNEEDTENLKWLQIFNKYDLYSKSKVRIDVEKVKPYYLSLTEKYFPA
GdMIOX07       ...LHKSGAYRHLMNEEDTENLKWLQIFNKYDLYSKSKVRIDVEKVKPYYLSLTEKYFPA
GtMIOX07       ...LHKSGAYRHLMNEEDTENLKWLQIFNKYDLYSKSKVRIDVEKVKPYYLSLIEKYFPA
GmMIOX07       ...LHKSGAYRHLMNEEDTENLKWLQIFNKYDLYSKSKVRIDVEKVKPYYLSLIEKYFPA
GherMIOX02     ...LHKSGAYRHLMNEEDTENMKWLQIFNKYDLYSKSKVRIDVEKVKPYYLSLIEKVGTT
GkkMIOX02      ...LHKSGAYRHLMNEEDTENLKWLQIFNKYDLYSKSKVRIDVEKVKPYYLPLIEKVGTT
GdMIOX02       ...LHKSGAYRHLMNEEDTENMKWLQIFNKYDLYSKSKVRIDVEKVKPYYLSLIEKYFPA
ATMIOX01       ...LHKSEAYKHLMNNEDRENMKWLKVFNKYDLYSKSKVRVNVEEVKPYYLSLTNKYFPS
GaMIOX06       ...LHRCGAYMHLMNEEDIENLKWLKIFNKYDLYSKSKVHIHVEKVKPYYLSFINKYFPA
GhMIOX05       ...LHRCGAYMHLMNEEDIENLKWLKIFNKYDLYSKSKVHIHVEKVKPYYLSLINKYFPA
GbMIOX11       ............................................................
GdMIOX10       ........AYMHLINEEDIENLKWLKIFNKFDLYSKSKVHIRVEKVKPYYLYLINKYFPA
GtMIOX10       ........AYMHLINEEDIENLKWLKIFNKFDLYSKSKVHIRVEKVKPYYLYLINKYFPA
GrMIOX02       ...HFRCGAYMHLINEEDIENLKWLKIFNKFDLYSKSKVHIRVEKVKPYYLYLINKYFPA
GhMIOX11       ...KFRCGAYMHLINEEDIENLKWLKIFNKFDLYSKSKVHIRVEKVKPYYLYLINKYFPA
GmMIOX10       ........AYMHLINEEDIENLKWLKIFNKFDLYSKSKVHIRVEKVKPYYLYLINKYFPA
GbMIOX05       ............................................................
GherMIOX05     EVGEALNEAFKGSLKREDIFLTSKLYCTMNSLNKIENYVRVSLKNLGVSYLDLYLMHWPE
GkkMIOX06      FYALHRCGAYKHLMNEEDIENLKWLMIFK...............................
```

```
            300
GdMIOX01    KLKW........................................................
GtMIOX01    KLKW........................................................
GbMIOX01    KLKW........................................................
GaMIOX01    KLKW........................................................
GkkMIOX01   ............................................................
GhMIOX01    KLKW........................................................
GmMIOX01    KLKW........................................................
GherMIOX01  KLKW........................................................
GbMIOX07    ............................................................
GtMIOX06    KLKW........................................................
GrMIOX01    KLKW........................................................
GhMIOX07    KLKW........................................................
GdMIOX06    ............................................................
GmMIOX06    KLKW........................................................
GmMIOX05    KLKW........................................................
GtMIOX05    KLKW........................................................
GdMIOX05    KLKW........................................................
GaMIOX05    KLKW........................................................
GbMIOX06    KLKW........................................................
GhMIOX06    KLKW........................................................
GhMIOX12    KLKW........................................................
GtMIOX11    KLKW........................................................
GbMIOX12    KLKW........................................................
GrMIOX03    KLKW........................................................
GherMIOX06  KLKW........................................................
GdMIOX11    KLKW........................................................
GmMIOX11    KLKW........................................................
GkkMIOX05   ............................................................
ATMIOX03    NLRW........................................................
ATMIOX04    NLRW........................................................
GaMIOX03    KLKW........................................................
GdMIOX03    KLKW........................................................
GherMIOX03  KLKW........................................................
GbMIOX03    KLKW........................................................
GtMIOX03    KLKW........................................................
GhMIOX03    KLKW........................................................
GmMIOX03    KLKW........................................................
GkkMIOX03   ............................................................
GtMIOX08    ............................................................
GbMIOX09    KLKW........................................................
GdMIOX08    KLKW........................................................
GhMIOX09    KLKW........................................................
GrMIOX05    KLKW........................................................
GmMIOX08    KLKW........................................................
ATMIOX02    KLKW........................................................
GherMIOX04  MAGEYPRSVLIALVIFSLVVSPMLPCVTARPPVVCPACVCCGPPPPGGACCSCGCASVQS
GhMIOX04    KLRW........................................................
GaMIOX04    KLRW........................................................
GbMIOX04    KLRW........................................................
GtMIOX04    KLRW........................................................
GmMIOX04    KLRW........................................................
GdMIOX09    KLRW........................................................
GtMIOX09    KLRW........................................................
GbMIOX10    KLRW........................................................
GhMIOX10    KLRW........................................................
GmMIOX09    KLRW........................................................
GrMIOX06    KLRW........................................................
GdMIOX04    KLRW........................................................
GkkMIOX04   KLRW........................................................
GhMIOX02    KLRW........................................................
GtMIOX02    KLRW........................................................
GbMIOX02    KLRW........................................................
GmMIOX02    KLRW........................................................
GaMIOX02    KLRW........................................................
GhMIOX08    ............................................................
GrMIOX04    KLRW........................................................
GbMIOX08    KLRW........................................................
GdMIOX07    KLRW........................................................
GtMIOX07    KLRW........................................................
GmMIOX07    KLRW........................................................
GherMIOX02  ILPGEVKMVNPGFSPSRQFNASASGFF.................................
GkkMIOX02   ILPGEVKMVNPGLSPSRRFNASASGFV.................................
GdMIOX02    KLRW........................................................
ATMIOX01    KLKW........................................................
GaMIOX06    KLNWRCGS....................................................
GhMIOX05    KLNWRCGS....................................................
GbMIOX11    ............................................................
GdMIOX10    KLKWQCGS....................................................
GtMIOX10    KLKWQCGS....................................................
GrMIOX02    KLKWQCGS....................................................
GhMIOX11    KLKWQCGS....................................................
GmMIOX10    KLKWQCGS....................................................
GbMIOX05    ............................................................
GherMIOX05  SSGFGDATDPPLKSGSEHRQFLNRLKKVWKAMEGLVDSGLVRAIGVSNFGVHQIKELLKF
GkkMIOX06   ............................................................
```

```
GdMIOX01     ............................................................
GtMIOX01     ............................................................
GbMIOX01     ............................................................
GaMIOX01     ............................................................
GkkMIOX01    ............................................................
GhMIOX01     ............................................................
GmMIOX01     ............................................................
GherMIOX01   ............................................................
GbMIOX07     ............................................................
GtMIOX06     ............................................................
GrMIOX01     ............................................................
GhMIOX07     ............................................................
GdMIOX06     ............................................................
GmMIOX06     ............................................................
GmMIOX05     ............................................................
GtMIOX05     ............................................................
GdMIOX05     ............................................................
GaMIOX05     ............................................................
GbMIOX06     ............................................................
GhMIOX06     ............................................................
GhMIOX12     ............................................................
GtMIOX11     ............................................................
GbMIOX12     ............................................................
GrMIOX03     ............................................................
GherMIOX06   ............................................................
GdMIOX11     ............................................................
GmMIOX11     ............................................................
GkkMIOX05    ............................................................
ATMIOX03     ............................................................
ATMIOX04     ............................................................
GaMIOX03     ............................................................
GdMIOX03     ............................................................
GherMIOX03   ............................................................
GbMIOX03     ............................................................
GtMIOX03     ............................................................
GhMIOX03     ............................................................
GmMIOX03     ............................................................
GkkMIOX03    ............................................................
GtMIOX08     ............................................................
GbMIOX09     ............................................................
GdMIOX08     ............................................................
GhMIOX09     ............................................................
GrMIOX05     ............................................................
GmMIOX08     ............................................................
ATMIOX02     ............................................................
GherMIOX04   PPSEMATP....................................................
GhMIOX04     ............................................................
GaMIOX04     ............................................................
GbMIOX04     ............................................................
GtMIOX04     ............................................................
GmMIOX04     ............................................................
GdMIOX09     ............................................................
GtMIOX09     ............................................................
GbMIOX10     ............................................................
GhMIOX10     ............................................................
GmMIOX09     ............................................................
GrMIOX06     ............................................................
GdMIOX04     ............................................................
GkkMIOX04    ............................................................
GhMIOX02     ............................................................
GtMIOX02     ............................................................
GbMIOX02     ............................................................
GmMIOX02     ............................................................
GaMIOX02     ............................................................
GhMIOX08     ............................................................
GrMIOX04     ............................................................
GbMIOX08     ............................................................
GdMIOX07     ............................................................
GtMIOX07     ............................................................
GmMIOX07     ............................................................
GherMIOX02   ............................................................
GkkMIOX02    ............................................................
GdMIOX02     ............................................................
ATMIOX01     ............................................................
GaMIOX06     ............................................................
GhMIOX05     ............................................................
GbMIOX11     ............................................................
GdMIOX10     ............................................................
GtMIOX10     ............................................................
GrMIOX02     ............................................................
GhMIOX11     ............................................................
GmMIOX10     ............................................................
GbMIOX05     ............................................................
GherMIOX05   AKIVPAVNQVELHPFWRQDELVKFCQMKGIHVSAHTPLGVPTSSPGVSDSGSGGEDEPGT
GkkMIOX06    ............................................................
```

```
GdMIOX01     ............................................................
GtMIOX01     ............................................................
GbMIOX01     ............................................................
GaMIOX01     ............................................................
GkkMIOX01    ............................................................
GhMIOX01     ............................................................
GmMIOX01     ............................................................
GherMIOX01   ............................................................
GbMIOX07     ............................................................
GtMIOX06     ............................................................
GrMIOX01     ............................................................
GhMIOX07     ............................................................
GdMIOX06     ............................................................
GmMIOX06     ............................................................
GmMIOX05     ............................................................
GtMIOX05     ............................................................
GdMIOX05     ............................................................
GaMIOX05     ............................................................
GbMIOX06     ............................................................
GhMIOX06     ............................................................
GhMIOX12     ............................................................
GtMIOX11     ............................................................
GbMIOX12     ............................................................
GrMIOX03     ............................................................
GherMIOX06   ............................................................
GdMIOX11     ............................................................
GmMIOX11     ............................................................
GkkMIOX05    ............................................................
ATMIOX03     ............................................................
ATMIOX04     ............................................................
GaMIOX03     ............................................................
GdMIOX03     ............................................................
GherMIOX03   ............................................................
GbMIOX03     ............................................................
GtMIOX03     ............................................................
GhMIOX03     ............................................................
GmMIOX03     ............................................................
GkkMIOX03    ............................................................
GtMIOX08     ............................................................
GbMIOX09     ............................................................
GdMIOX08     ............................................................
GhMIOX09     ............................................................
GrMIOX05     ............................................................
GmMIOX08     ............................................................
ATMIOX02     ............................................................
GherMIOX04   ............................................................
GhMIOX04     ............................................................
GaMIOX04     ............................................................
GbMIOX04     ............................................................
GtMIOX04     ............................................................
GmMIOX04     ............................................................
GdMIOX09     ............................................................
GtMIOX09     ............................................................
GbMIOX10     ............................................................
GhMIOX10     ............................................................
GmMIOX09     ............................................................
GrMIOX06     ............................................................
GdMIOX04     ............................................................
GkkMIOX04    ............................................................
GhMIOX02     ............................................................
GtMIOX02     ............................................................
GbMIOX02     ............................................................
GmMIOX02     ............................................................
GaMIOX02     ............................................................
GhMIOX08     ............................................................
GrMIOX04     ............................................................
GbMIOX08     ............................................................
GdMIOX07     ............................................................
GtMIOX07     ............................................................
GmMIOX07     ............................................................
GherMIOX02   ............................................................
GkkMIOX02    ............................................................
GdMIOX02     ............................................................
ATMIOX01     ............................................................
GaMIOX06     ............................................................
GhMIOX05     ............................................................
GbMIOX11     ............................................................
GdMIOX10     ............................................................
GtMIOX10     ............................................................
GrMIOX02     ............................................................
GhMIOX11     ............................................................
GmMIOX10     ............................................................
GbMIOX05     ............................................................
GherMIOX05   PRISFRRSRSVHGPMLKLSVVGEIADRHKKTPEQVILRWGFQRGTSVLPCSLKPDRIKQN
GkkMIOX06    ............................................................
```

```
GdMIOX01     ............................................................
GtMIOX01     ............................................................
GbMIOX01     ............................................................
GaMIOX01     ............................................................
GkkMIOX01    ............................................................
GhMIOX01     ............................................................
GmMIOX01     ............................................................
GherMIOX01   ............................................................
GbMIOX07     ............................................................
GtMIOX06     ............................................................
GrMIOX01     ............................................................
GhMIOX07     ............................................................
GdMIOX06     ............................................................
GmMIOX06     ............................................................
GmMIOX05     ............................................................
GtMIOX05     ............................................................
GdMIOX05     ............................................................
GaMIOX05     ............................................................
GbMIOX06     ............................................................
GhMIOX06     ............................................................
GhMIOX12     ............................................................
GtMIOX11     ............................................................
GbMIOX12     ............................................................
GrMIOX03     ............................................................
GherMIOX06   ............................................................
GdMIOX11     ............................................................
GmMIOX11     ............................................................
GkkMIOX05    ............................................................
ATMIOX03     ............................................................
ATMIOX04     ............................................................
GaMIOX03     ............................................................
GdMIOX03     ............................................................
GherMIOX03   ............................................................
GbMIOX03     ............................................................
GtMIOX03     ............................................................
GhMIOX03     ............................................................
GmMIOX03     ............................................................
GkkMIOX03    ............................................................
GtMIOX08     ............................................................
GbMIOX09     ............................................................
GdMIOX08     ............................................................
GhMIOX09     ............................................................
GrMIOX05     ............................................................
GmMIOX08     ............................................................
ATMIOX02     ............................................................
GherMIOX04   ............................................................
GhMIOX04     ............................................................
GaMIOX04     ............................................................
GbMIOX04     ............................................................
GtMIOX04     ............................................................
GmMIOX04     ............................................................
GdMIOX09     ............................................................
GtMIOX09     ............................................................
GbMIOX10     ............................................................
GhMIOX10     ............................................................
GmMIOX09     ............................................................
GrMIOX06     ............................................................
GdMIOX04     ............................................................
GkkMIOX04    ............................................................
GhMIOX02     ............................................................
GtMIOX02     ............................................................
GbMIOX02     ............................................................
GmMIOX02     ............................................................
GaMIOX02     ............................................................
GhMIOX08     ............................................................
GrMIOX04     ............................................................
GbMIOX08     ............................................................
GdMIOX07     ............................................................
GtMIOX07     ............................................................
GmMIOX07     ............................................................
GherMIOX02   ............................................................
GkkMIOX02    ............................................................
GdMIOX02     ............................................................
ATMIOX01     ............................................................
GaMIOX06     ............................................................
GhMIOX05     ............................................................
GbMIOX11     ............................................................
GdMIOX10     ............................................................
GtMIOX10     ............................................................
GrMIOX02     ............................................................
GhMIOX11     ............................................................
GmMIOX10     ............................................................
GbMIOX05     ............................................................
GherMIOX05   IDIFNWSLSDDEWNRLNRIEPQVCLYGDGPLNNLSDRGFMFGSGPLQAVREMEDDAEFNA
GkkMIOX06    ............................................................
```
