# Supplementary figures and images for: Genome-wide identification of the MIOX gene family and their expression profile in cotton development and response to abiotic stress
Source: PLoS One. 2021 Jul 9;16(7):e0254111. doi: 10.1371/journal.pone.0254111 (PMC8270170; doi:10.1371/journal.pone.0254111)

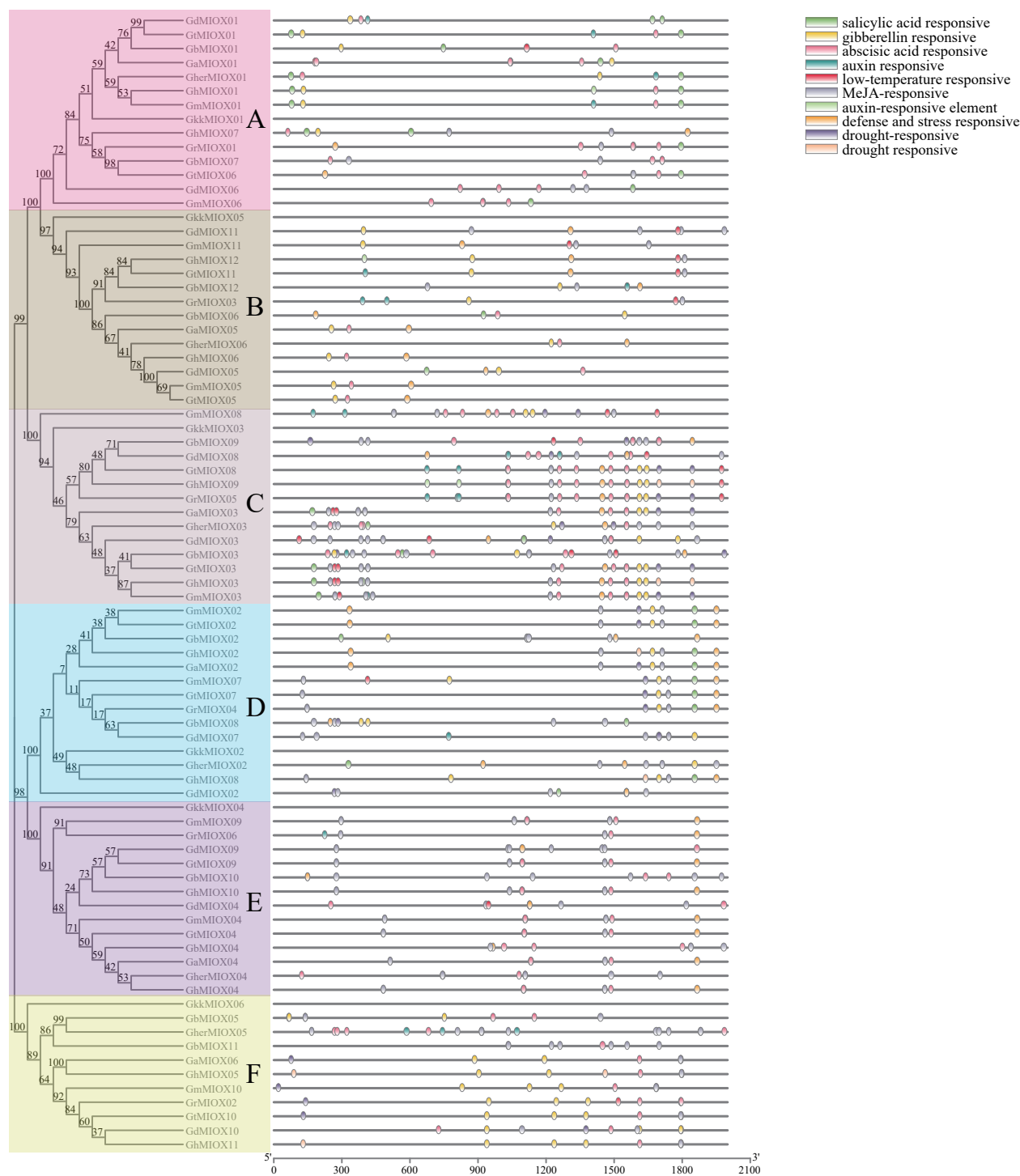

Supplement: S2 Fig — The promoter sequences (2 kb upstream of ATG) of the MIOX genes were analysed by PlantCARE. (PDF) [file pone.0254111.s002.pdf]
